# A Novel Multiple Risk Score Model for Prediction of Long-Term Ischemic Risk in Patients With Coronary Artery Disease Undergoing Percutaneous Coronary Intervention: Insights From the I-LOVE-IT 2 Trial

**DOI:** 10.3389/fcvm.2021.756379

**Published:** 2022-01-13

**Authors:** Miaohan Qiu, Yi Li, Kun Na, Zizhao Qi, Sicong Ma, He Zhou, Xiaoming Xu, Jing Li, Kai Xu, Xiaozeng Wang, Yaling Han

**Affiliations:** ^1^Second Affiliated Hospital of Dalian Medical University, Dalian, China; ^2^The Department of Cardiology, General Hospital of Northern Theater Command, Shenyang, China; ^3^Postgraduate College, Shenyang Pharmaceutical University, Shenyang, China; ^4^Second Affiliated Hospital of Harbin Medical University, Harbin, China; ^5^The Second Hospital of Jilin University, Changchun, China; ^6^Postgraduate College, Liaoning University of Traditional Chinese Medicine, Shenyang, China

**Keywords:** coronary artery disease, percutaneous coronary intervention, risk score, ischemic events, drug-eluting stent

## Abstract

**Backgrounds:** A plug-and-play standardized algorithm to identify the ischemic risk in patients with coronary artery disease (CAD) undergoing percutaneous coronary intervention (PCI) could play a valuable step to help a wide spectrum of clinic workers. This study intended to investigate the ability to use the accumulation of multiple clinical routine risk scores to predict long-term ischemic events in patients with CAD undergoing PCI.

**Methods:** This was a secondary analysis of the I-LOVE-IT 2 (Evaluate Safety and Effectiveness of the Tivoli drug-eluting stent (DES) and the Firebird DES for Treatment of Coronary Revascularization) trial, which was a prospective, multicenter, and randomized study. The Global Registry for Acute Coronary Events (GRACE), baseline Synergy Between Percutaneous Coronary Intervention with Taxus and Cardiac Surgery (SYNTAX), residual SYNTAX, and age, creatinine, and ejection fraction (ACEF) score were calculated in all patients. Risk stratification was based on the number of these four scores that met the established thresholds for the ischemic risk. The primary end point was ischemic events at 48 months, defined as the composite of cardiac death, nonfatal myocardial infarction, stroke, or definite/probable stent thrombosis (ST).

**Results:** The 48-month ischemic events had a significant trend for higher event rates (from 6.61 to 16.93%) with an incremental number of risk scores presenting the higher ischemic risk from 0 to ≥3 (*p* trend < 0.001). In addition, the categories were associated with increased risk for all components of ischemic events, including cardiac death (from 1.36 to 3.15%), myocardial infarction (MI) (from 3.31 to 9.84%), stroke (3.31 to 6.10%), definite/probable ST (from 0.58 to 1.97%), and all-cause mortality (from 2.14 to 6.30%) (all *p* trend < 0.05). The net reclassification index after combined with four risk scores was 12.5% (5.3–20.0%), 9.4% (2.0–16.8%), 12.1% (4.5–19.7%), and 10.7% (3.3–18.1%), which offered statistically significant improvement in the performance, compared with SYNTAX, residual SYNTAX, ACEF, and GRACE score, respectively.

**Conclusion:** The novel multiple risk score model was significantly associated with the risk of long-term ischemic events in these patients with an increment of scores. A meaningful improvement to predict adverse outcomes when multiple risk scores were applied to risk stratification.

## Introduction

Personalized medicine is a medical model that separates patients into different groups with tailored medical decisions, practices, and interventions based on their predicted risk of disease. Theoretically, taking a series of risk factors into account to evaluate the individual risk of patients with coronary artery disease (CAD) undergoing percutaneous coronary intervention (PCI) before the decision-making process was superior to “one-size-fits-all” approaches (1–3).

Recently a variety of risk scoring systems, as comprehensive predicted tools for risk assessment, have been developed to support physicians in clinical practice for these patients (4–7). However, to our knowledge, there was not a robust, interoperable, and universal risk score that could be extended to different populations, which is mainly caused by prediction algorithms derived from different cohorts and a complex and time-varying clinical process (8–11). Meanwhile, previous studies demonstrate that an additive value of one risk score combined with a biomarker, angiographic characteristic, and with another risk score to risk predicting (12–14).

Thus, we sought to investigate whether using a strategy assisted by the accumulation of multiple clinical routine risk algorithms could improve the ability of discrimination to predict long-term ischemic events in patients with CAD undergoing PCI.

## Materials and Methods

### Study Design and Patients

This was a secondary analysis of the I-LOVE-IT 2 (Evaluate Safety and Effectiveness of the Tivoli drug-eluting stent (DES) and the Firebird DES for Treatment of Coronary Revascularization; NCT01681381) trial, which was a prospective, multicenter, randomized, assessor-blinded, and non-inferiority study that compared a biodegradable polymer sirolimus-eluting stent (BP-SES, Tivoli, Essen Tech, Beijing, China) with a durable polymer sirolimus-eluting stent (DP-SES, Firebird 2, MicroPort, Shanghai, China). Study details have been previously described (15, 16). In brief, between October 2012 and June 2013, a total of 2,737 patients presenting with stable CAD or acute coronary syndromes (ACS) were randomly assigned to undergo PCI with either BP-SES or DP-SES at a 2:1 ratio at 32 centers in China. Patients who were randomized to the BP-SES group were additionally re-randomized to a 6- or 12-month DAPT group at a 1:1 ratio. All patients were discharged with a prescription for at least 100 mg aspirin indefinitely and 75 mg clopidogrel for 6 or 12 months after stent implantation. Qualitative and quantitative coronary angiography (including Synergy between Percutaneous Coronary Intervention with Taxus and Cardiac Surgery (SYNTAX) score and residual SYNTAX score) were centrally evaluated by a blinded independent core laboratory (CCRF, Beijing, China) using QAngio XA Version 7.3 Analysis Software (Medis Medical Imaging System, Leiden, The Netherlands). The study complies with the provisions of the Declaration of Helsinki, and the study protocol was approved by the institutional review board at each participating site. All patients provided written informed consent.

### Risk Assessment

For these analyses, four risk scores, which were supported by an extensive, rigorous external validation process, and/or endorsed by current guidelines, were used to predict the ischemic risk after PCI, as follows. (1) Discharge Global Registry for Acute Coronary Events (GRACE) score (17) was calculated and described based on age, history of congestive heart failure, history of myocardial infarction (MI), resting heart rate, systolic blood pressure, ST-segment depression, initial serum creatinine, elevated cardiac enzymes, and PCI in-hospital. Patients were considered intermediate to high ischemic risk for scores ≥88 (18). (2) Baseline SYNTAX score is a comprehensive angiographic scoring system that is derived entirely from the coronary anatomy and lesion characteristics, which was designed to quantify lesion complexity before the procedure (19). The baseline SYNTAX Score may aid in assessing the ischemic events, including cardiac death, MI, and target vessel revascularization (20). The baseline SYNTAX score value of 13 is considered an optimal cutoff point depending on the prognosis of patients (20). (3) Age, creatinine, and ejection fraction (ACEF) score developed by Ranucci et al. (21) was a simple tool for predicting in-hospital mortality in patients undergoing elective cardiac surgery. Meanwhile, a previous study showed that the ACEF score had a good discriminative in patients undergoing PCI (22). The ACEF score ≥1.0225 might be useful and applicable for risk stratification in these populations with respect to the long-term clinical prognosis (22). (4) Residual SYNTAX score (rSS) was first proposed by Généreux et al. (23), which was calculated based on the remaining obstructive coronary disease after treatment with PCI. The rSS could be used to quantify the burden and complexity of residual CAD after the procedure. The rSS of >0 was associated with long-term ischemic outcomes, including all-cause mortality and MI (24, 25). Risk score calculations are shown in Supplementary Appendix S1.

The method of risk stratification in the current study was calculated using the number of these four scores (called ACE-SYNTAX score) that met the thresholds for the intermediate- or high-risk, ranging from 0 to ≥3, logically a total of four categories, in all patients.

### Outcomes

All clinical and laboratory variables included in the present analysis were prospectively collected. The multiple risk score model was developed to predict ischemic events at 48 months, defined as the composite of cardiac death, nonfatal MI, stroke, or definite/probable stent thrombosis (ST). The definitions of those endpoints were described in the previous report (15, 16). All patients were followed-up with by telephone or hospital visits at 1, 6, 9, 12, and 18 months, and annually for up to 5 years. All clinical events were adjudicated by a blinded independent clinical events committee.

### Statistical Analysis

Patient characteristics were stratified according to the risk stratification of risk scores. Continuous variables are presented as mean ± SD; categorical variables are displayed as counts and percentages. Comparisons were performed with a chi-square test for categorical variables and one-way ANOVA for continuous variables. Testing for trends in event rates across risk scores was done with the Cochran–Armitage test. Time-to-event data with estimated event rates measured with the Kaplan–Meier method were compared using the log-rank test. An individual risk score was evaluated for the discrimination for 4-year ischemic events. The discrimination of individual risk score was measured by the receiver operator characteristic curve (ROC) with the area under the curve (AUC), which ranges from 0.50 (no discrimination) to 1.0 (perfect discrimination). The net reclassification improvement (NRI) analysis was performed to assess the improved ability of combined risk scores for risk stratification over the single score (26). To deal with the missing components of the risk scores that occurred at random, multiple imputations were performed using chained equations. Missing values were predicted on the basis of all other clinical variables. The Cox regression estimates from each imputed dataset were averaged together to produce an overall hazard ratio (*HR*) computed using the Rubin rule. Unless otherwise specified, a 2-sided *p*-value of < 0.05 was considered to indicate statistical significance. Statistical analysis was performed using SAS software version 9.3 (SAS Institute, Cary, NC, USA).

## Results

A total of 2,207 patients with 3,027 lesions were selected and calculated ACE-SYNTAX score and were analyzed in the study. ACEF score was not fully evaluable in 342 patients due to the missing data of ejection fraction (240 cases) or creatinine clearance (102 cases). Meanwhile, the GRACE score could not be calculated in 188 cases with a lack of cardiac enzymes. The outcomes of these 530 patients excluded from the analysis are shown in Supplementary Table S1.

The baseline SYNTAX score ranged from 0 to 55, with a mean ± SD of 11.9 ± 8.3, and a median of 10.0 (6.0–16.0). The residual SYNTAX score ranged from 0 to 53, with a mean ± SD of 3.4 ± 5.2, and a median of 0.0 (0.0–5.0). The ACEF score ranged from 0.4 to 7.6, with a mean ± SD of 1.3 ± 0.8, and a median of 1.0 (0.9–1.2). The GRACE score ranged from 16 to 153, with a mean ± SD of 76.0 ± 21.5, and a median of 77.0 (60.0–91.0). By using the previously validated cutoffs described in the methods, 831 patients (37.65%) based on baseline SYNTAX score, 1,053 patients (47.71%) based on residual SYNTAX score, 995 patients (45.08%) based on ACEF score, and 650 patients (29.45%) based on GRACE score met the thresholds for the intermediate or high-risk category. A Venn diagram was shown to demonstrate the coexistence of conditions of these risk scores (Supplementary Figure S1).

Among 2,207 patients, the risk score of 514 (23.3%) patients who failed to reach any of the four scores cutoff value was defined as zero. The number of other groups, 1 to ≥3 risk-score, were 553 (25.1%), 632 (28.6%), and 508(23.0%), respectively. The overall distribution of incremental risk-score categories was displayed in Supplementary Figure S2. The baseline demographics and calculation of risk scores are reported in Table 1. The antiplatelet therapy during the follow-up period is shown in Supplementary Table S2. The lesion characteristics and procedural results are shown in Table 2 stratified across cumulative risk-score categories.

**Table 1:** Baseline demographics and score calculation stratified across cumulative risk-score categories.

	**No. of risk scores met the individual thresholds**				***P*-value**
	**0 (*N* = 514)**	**1 (*N* = 553)**	**2 (*N* = 632)**	**≥3 (*N* = 508)**	
Age, yrs	53.20 ± 7.89	57.32 ± 8.07	61.80 ± 9.89	68.24 ± 7.34	<0.001
Men, No. (%)	382 (74.32%)	393 (71.07%)	426 (67.41%)	320 (62.99%)	<0.001
Body mass index, mean ± SD	25.67 ± 3.15	25.31 ± 2.87	25.24 ± 3.15	24.77 ± 3.05	<0.001
Diabetes mellitus	108 (21.01%)	115 (20.80%)	145 (22.94%)	136 (26.77%)	0.08
Hypertension	305 (59.34%)	338 (61.12%)	402 (63.61%)	355 (69.88%)	0.003
Hyperlipidemia	140 (27.24%)	151 (27.31%)	143 (22.63%)	117 (23.03%)	0.12
Peripheral arterial disease	3 (0.58%)	6 (1.08%)	6 (0.95%)	9 (1.77%)	0.32
Previous myocardial infarction	55 (10.70%)	82 (14.83%)	118 (18.67%)	112 (22.05%)	<0.001
Previous stroke	30 (5.84%)	62 (11.21%)	75 (11.87%)	57 (11.22%)	0.003
Previous PCI	24 (4.67%)	49 (8.86%)	64 (10.13%)	32 (6.30%)	0.002
Previous CABG	1 (0.19%)	2 (0.36%)	3 (0.47%)	6 (1.18%)	0.15
Smoking history					0.006
Current smoker	223 (43.39%)	222 (40.14%)	228 (36.08%)	283 (55.71%)	
Ex-smoker	55 (10.70%)	64 (11.57%)	73 (11.55%)	159 (31.30%)	
None	236 (45.91%)	267 (48.28%)	331 (52.37%)	66 (12.99%)	
Family history of CAD	41 (7.98%)	44 (7.96%)	35 (5.54%)	23 (4.53%)	
Type of CAD, No. (%)					<0.001
STEMI	47 (9.14%)	62 (11.21%)	89 (14.08%)	78 (15.35%)	
NSTEMI	42 (8.17%)	47 (8.50%)	64 (10.13%)	78 (15.35%)	
Unstable angina	341 (66.34%)	341 (61.66%)	371 (58.70%)	281 (55.31%)	
Others	84 (16.34%)	103 (18.63%)	108 (17.09%)	71 (13.98%)	
Ccr, mean ± SD	110.67 ± 30.00	99.69 ± 32.30	91.36 ± 30.82	72.66 ± 23.67	<0.001
LVEF, %	64.01 ± 5.87	61.54 ± 7.85	60.37 ± 8.38	56.72 ± 8.72	<0.001
Anemia	9 (1.77%)	20 (3.67%)	28 (4.47%)	47 (9.36%)	<0.001
**Risk scores**					
Baseline SYNTAX score	5.71 ± 3.15	9.50 ± 6.62	13.70 ± 8.25	18.38 ± 8.00	<0.001
Residual SYNTAX score	0.00 ± 0.00	1.65 ± 2.39	4.61 ± 5.65	7.02 ± 6.56	<0.001
ACEF score	0.83 ± 0.12	1.06 ± 0.52	1.31 ± 0.82	1.80 ± 1.09	<0.001
GRACE score	60.31 ± 14.80	68.54 ± 16.33	79.57 ± 20.64	95.41 ± 16.25	<0.001

*Values are mean ± SD or No. (%). RS, risk score; BMI, body mass index; PCI, percutaneous coronary intervention; CABG, coronary artery bypass graft; CAD, coronary artery disease; STEMI, ST-segment-elevation myocardial infarction; NSTEMI, non-ST-segment-elevation myocardial infarction; Ccr, creatinine clearance; LVEF, left ventricular ejection fraction; SYNTAX, synergy between PCI with TAXUS and cardiac surgery; ACEF, age, creatinine and ejection fraction; GRACE, global registry for acute coronary events*.

**Table 2:** Lesion characteristics and procedural results stratified across cumulative risk-score categories.

	**No. of risk scores met the individual thresholds**				***P*-value**
	**0 (514 Patients, 625 Lesions)**	**1 (553 Patients, 757 Lesions)**	**2 (632 Patients, 891 Lesions)**	**≥3 (508 Patients, 774 Lesions)**	
Transradial approach	486 (94.55%)	521 (94.21%)	577 (91.30%)	468 (92.13%)	0.09
Target vessel disease extent					<0.001
1-vessel	430 (83.66%)	406 (73.42%)	439 (69.46%)	311 (61.22%)	
2-vessel	63 (12.26%)	123 (22.24%)	162 (25.63%)	149 (29.33%)	
3-vessel	2 (0.39%)	12 (2.17%)	14 (2.22%)	23 (4.53%)	
Left main artery	19 (3.70%)	12 (2.17%)	17 (2.69%)	25 (4.92%)	
Baseline SYNTAX score	5.71 ± 3.15	9.50 ± 6.62	13.70 ± 8.25	18.38 ± 8.00	<0.001
No. of target lesions per patient	1.22 ± 0.43	1.37 ± 0.60	1.41 ± 0.60	1.52 ± 0.68	<0.001
Target vessel location					0.16
Left main artery	19 (3.04%)	12 (1.59%)	17 (1.91%)	26 (3.36%)	
Left anterior descending artery	284 (45.44%)	324 (42.86%)	417 (46.80%)	335 (43.28%)	
Left circumflex artery	142 (22.72%)	183 (24.21%)	183 (20.54%)	173 (22.35%)	
Right coronary artery	180 (28.80%)	237 (31.35%)	274 (30.75%)	240 (31.01%)	
ACC/AHA lesion classification B2+C	501 (80.16%)	635 (83.99%)	757 (84.96%)	685 (88.50%)	<0.001
Bifurcation lesion	181 (28.96%)	235 (31.08%)	280 (31.43%)	317 (40.96%)	<0.001
Total occlusion	42 (6.72%)	75 (9.92%)	127 (14.25%)	116 (14.99%)	<0.001
Severely tortuous or angulated lesion	11 (1.76%)	13 (1.72%)	24 (2.69%)	28 (3.62%)	0.06
Moderate to heavy calcification	13 (2.08%)	15 (1.98%)	27 (3.03%)	41 (5.30%)	<0.001
**Pre-procedural QCA**					
Reference vessel diameter, mm	2.84 ± 0.49	2.82 ± 0.48	2.76 ± 0.46	2.74 ± 0.43	<0.001
Lesion length, mm	18.62 ± 11.55	19.86 ± 11.16	21.78 ± 12.98	23.66 ± 14.12	<0.001
**Procedural results**					
Stent per patient	1.51 ± 0.70	1.67 ± 0.83	1.81 ± 0.87	2.02 ± 0.96	<0.001
Total stent length per patient, mm	35.70 ± 20.16	40.42 ± 22.90	44.64 ± 25.20	51.17 ± 27.03	<0.001
Residual SYNTAX score	0.00 ± 0.00	1.65 ± 2.39	4.61 ± 5.65	7.02 ± 6.56	<0.001

*Values are mean ± SD or No. (%). ACC/AHA, American College of Cardiology/American Heart Association; QCA, quantitative coronary angiography; Other abbreviations are the same as for Table 1*.

### Stratified Clinical Outcomes

The 48-month ischemic events had a significant trend for higher event rates (from 6.61 to 16.93%) with incremental risk-score categories from 0 to ≥3 (*p*_trend_ < 0.001). The categories were also associated with increased risk for all components of ischemic events, including cardiac death (from 1.36 to 3.15%, *p*_trend_ = 0.025), all MI (from 3.31 to 9.84%, *p*_trend_ < 0.001), stroke (3.31 to 6.10%, *p*_trend_ = 0.013), definite/probable ST (from 0.58 to 1.97%, *p*_trend_ = 0.035), TVMI (from 2.92 to 8.66%, *p*_trend_ < 0.001), and all-cause mortality (from 2.14 to 6.30%, *p*_trend_ < 0.001) at 48 months with a significant trend according to risk-score categories (Table 3).

**Table 3:** Clinical outcomes at 4-year follow-up stratified across cumulative risk-score categories.

	**No. of risk scores met the individual thresholds**				P for trend^*^
	**0 (*N* = 514)**	**1 (*N* = 553)**	**2 (*N* = 632)**	**≥3 (*N* = 508)**	
Ischemic events	34 (6.61%)	51 (9.22%)	83 (13.13%)	86 (16.93%)	<0.001
All-cause mortality	11 (2.14%)	13 (2.35%)	21 (3.32%)	32 (6.30%)	<0.001
Cardiac death	7 (1.36%)	5 (0.90%)	8 (1.27%)	16 (3.15%)	0.025
All MI	17 (3.31%)	26 (4.70%)	41 (6.49%)	50 (9.84%)	<0.001
Target vessel MI	15 (2.92%)	20 (3.62%)	34 (5.38%)	44 (8.66%)	<0.001
Stroke	17 (3.31%)	23 (4.16%)	39 (6.17%)	31 (6.10%)	0.013
Definite/probable ST	3 (0.58%)	4 (0.72%)	5 (0.79%)	10 (1.97%)	0.035

*Values are No. (%). MI, myocardial infarction; ST, stent thrombosis. Ischemic events were defined as a composite of cardiac death, all MI, stroke, and /or definite/probable ST*.

* *Cochran-Armitage trend test*.

Using the multiple imputations for the missing values (ejection fraction, creatinine clearance, and cardiac enzymes), a total of 20 imputed datasets were generated. The trend of 48-month ischemic events was robust in each imputed dataset (all *p*_trend_ < 0.001, Supplementary Table S3).

**Figure 1:**
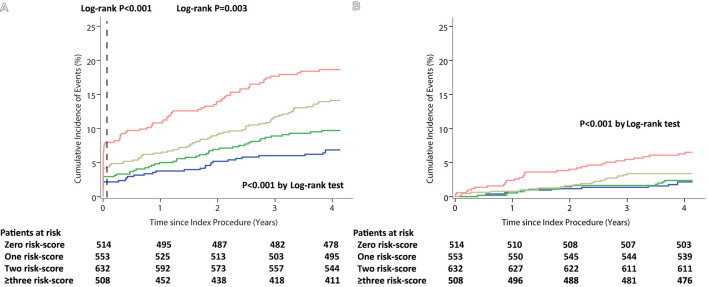
Kaplan–Meier curves during follow-up for 48-month Outcomes among the Cumulative Risk-score Categories. **(A)** Ischemic events (with 30-day landmark analysis). **(B)** All-cause Mortality.

There were consistent findings measured with the Kaplan–Meier method. The incidence of ischemic events, all-cause mortality at 4 years experienced a significant increase with the cumulative number of risk scores (both *p* < 0.001 by log-rank test). The landmark analysis showed that the patients with the higher cumulative risk-score were associated with a higher risk of ischemic events in the intervals of 0–30 days as well as 30 days to 4 years (from 2.1 to 7.68%, log-rank *p* < 0.001, and from 4.5 to 9.3%, log-rank *p* = 0.003, respectively) (Figure 1 and Supplementary Table S4). A sensitive analysis was performed and showed that the incidence of 48-month ischemic events had a consistent tendency with incremental risk-score categories with incremental risk-score categories (Supplementary Table S5).

**Figure 2:**
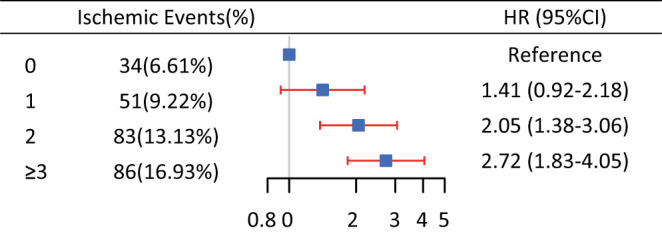
Incidence of ischemic events among cumulative risk-score categories.

It was shown that the 4-year rate of ischemic events was significantly higher in the patients with cumulative risk-score 2 to ≥3, leaving cumulative risk-score 0 as the reference [(*HR*: 2.05, 95% *CI*, 1.38–3.06), and (*HR*: 2.72, 95% CI, 1.83–4.05), respectively], whereas, this did not differ in patients with cumulative risk-scores 0 and 1 (*HR*: 1.41, 95% *CI*, 0.92–2.18) (Figure 2).

**Figure 3:**
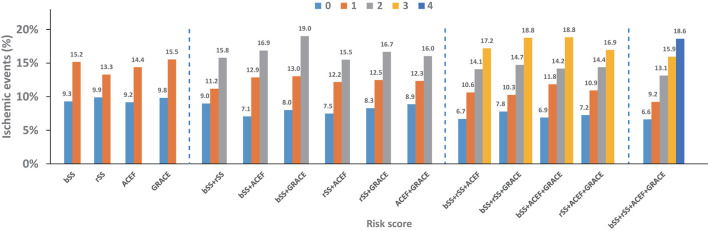
Four-year ischemic events stratified by individual and different combination of risk score (s).

The ischemic events at 48 months stratified by one and different combinations of risk score (s) are illustrated in Figure 3 and Supplementary Figure S3. Using cumulative risk-score categories could discriminate the risk of ischemia better than any single risk score, especially in patients with lower and higher ischemic risk. The combination with two risk scores of baseline SYNTAX and GRACE score has good discrimination to predict the 48-month ischemic events in all kinds of two risk scores. The combination with three risk scores of baseline SYNTAX, residual SYNTAX, and GRACE score and baseline SYNTAX, ACEF, and GRACE score has a better ability to assess the ischemic risk.

The ROC curves for 48-month ischemic events of the individual and an incremental number of risk scores, as continuous variables, are shown in Supplementary Figure S4. The discrimination of individual risk score was moderate, with AUC from 0.55 to 0.58. The AUC of an incremental number of risk scores was 0.61 (0.57–0.64). The best cutoffs of GRACE, baseline SYNTAX, ACEF, and residual SYNTAX score to predict 48-month ischemic events risk were 87, 12.5, 1.11, and 1 point(s), respectively. The optimal threshold of the ACE-SYNTAX model was two points for ischemic events at 48 months. Comparing with the baseline and residual SYNTAX score, the AUC of incremental number of risk scores at 48-month ischemic events had a significant improvement [0.57 (0.53–0.61), *p* = 0.038 and 0.55 (0.51–0.59), *p* = 0.001, respectively]. There was no significant improvement in AUC of ROC when compared ACEF and GRACE score with cumulative risk score [0.58 (0.54–0.62), *p* = 0.16 and 0.57 (0.54–0.61), *p* = 0.08, respectively]. Reclassification of patients into risk categories according to the occurrence of 48-month ischemic events is summarized in Supplementary Table S6. The NRI after combined with four risk scores was 12.5% (5.3–20.0%), 9.4% (2.0–16.8%), 12.1% (4.5–19.7%), and 10.7% (3.3–18.1%), which offers statistically significant improvement in the performance, compared with SYNTAX, residual SYNTAX, ACEF, and GRACE score, respectively.

## Discussion

The current study, which included data from a prospective, multicenter, and randomized trial, is the first study to investigate the feasibility and effectiveness of the management strategy (ACE-SYNTAX score) that combined with multiple risk scores could modify the discrimination to predict the long-term prognosis of patients with CAD undergoing PCI. The main findings of this analysis were as follows: (1) as the clinical routine risk scores, the baseline SYNTAX, residual SYNTAX, ACEF, and GRACE score demonstrated a certain value with respect to predicted long-term ischemic risk in CAD patients with stents implantation, with moderate discrimination; (2) the risk of ischemic events, including cardiac death, MI, stroke, definite/probable ST, and all-cause mortality have a significant increasing trend with incremental risk-score categories in these patients; and (3) using combinatory of predicting algorithms properly could play a valuable step to help clinicians identify the risk of these patients with implementation of sufficient treatments both in the post-procedure and long-term period, especially in these with lower or higher risk.

As we all know, the prognosis of patients with CAD is determined by baseline risk factors and the use of guideline-indicated therapies. The appropriately-stratified for these patients after stents implantation, which is a significant management challenge, has the potential to achieve the optimal individualized treatment and improve long-term outcomes (27–29). Thus, it is no doubt that using risk prediction algorithms to stratify the patients according to their estimated risk of future ischemic events could assist clinicians in selecting the optimal intensity and/or duration of secondary prevention therapy in decision-making. However, the gaps between guidelines and clinical practices were that the evidence-recommended tools to predict risk might be not universally applicable and robust. The main reason might be the timeliness of the risk scores, though it might be difficult to solve. The most frequently-used risk scores in our routine clinical practice, such as GRACE and SYNTAX scores, are developed from several years back in time. As the newest risk assessment tools, such as dual antiplatelet therapy (DAPT) and Predicting complications in patients undergoing stent implantation and subsequent Dual antiplatelet therapy (PRECISE-DAPT) scores, the data used to build them from randomized controlled trials or observational studies are still more than 5 years (4, 5). However, clinical practice and technology have advanced at a breathless pace. Over recent decades, medicine has drastically evolved with wider clinical use of more advanced diagnostic and therapeutic techniques, they might misestimate the risk of the disease (30). Meanwhile, the complex interaction between residual risk after PCI and the therapeutic benefit of secondary prevention management increases the complexity of risk stratification in these patients. Although no clinical risk tool is perfect, making use of the scores appropriately could provide convincing evidence in helping clinicians make individualized decisions for their patients. Utility of the accumulation of multiple risk scores could overcome the situation, having a significant improvement to discriminate risk of patients with a better classification.

Of note, evidence from a prior study suggests that medical management based on risk stratification was significantly associated with improved long-term prognosis, nevertheless, the benefits decreased with increasing estimated risk (31). The utilization of risk scores tends to be more successful in improving the reclassification of risk, which may enable monitoring and mobilizing clinical practice managers. Admittedly, there was no significant improvement in AUC of ROC at 48-month ischemic events when compared ACEF and GRACE scores with cumulative risk scores. However, it is intelligible that there is just integrating several validated risk scores without any other factors implantation. Still, the accumulative scores were proved a better net reclassification of risk compared with each single score. As we all know, adjustment of the weight of variables and bringing new factors into the risk score could increase the performance when the algorithm is insufficiently accurate in different races/ethnicity. Both above mentioned methods needed a series of cohorts to re-develop and re-validate the score. Our study demonstrates a plug-and-play strategy to risk assessment, which is especially suitable for these without established tools to carry out.

There is no escaping the fact that physicians routinely overestimated the risk of cardiac events and overvalued the benefits of invasive and secondary prevention management with a strong reliance on their intuition (32–34). Undoubtedly, predicting the adverse risk based on objectively quantified clinical algorithms could provide superior risk discrimination (32, 34). The number of prediction tools, as well as the presence of overlapping risk scores in the same clinical scenarios, is the blowout of a sharp increase, which makes it difficult to select using a universal and interoperable scoring system for the cardiologists. Indeed, in the clinical routine practice, using multiple complex algorithms could be challenging and cumbersome to compute.

However, with the improvement of the digital hospital and laboratory information system and the advent of machine learning based on deep neural networks, an approach may be a viable solution to generate detailed data in high-volume capacity (35, 36). It is convenient to capture all factors relating to the scores in the electronic medical records (EMR), calculate them automatically, and then quickly preset the predicting risk of patients in the system in auto (37, 38). It should be noted that dichotomizing continuous risk scores into a regression model might not be the optimal choice, which could induce a potential risk of inaccuracy. However, predictive accuracy for ischemic events was similar to continuation and dichotomization measurements in our research. Considering the clinical applicability without excessive consumption of accuracy, it might be acceptable to transfer continuous variables into binary variables.

The current study is limited by its *post-hoc* nature. As a retrospective analysis, the results of our study are hypothesis-generating. Thus, it is essential to confirm our findings in several specifically designed trials. Second, even with digital hospital and laboratory information systems, it is still complicated for clinicians to carry out too many risk-assessing tools. Therefore, in order to increase availability, what needs to be done further is investigating the proper combination of risk scores in different races/ethnicity. Third, the patients with missing data of risk scores were excluded in our study, which could have biased the estimates. Nevertheless, multiple imputations were performed to address it. The results were consistent between before and after imputation.

## Conclusion

The guideline-indicated ischemic risk scores displayed reasonable predictive performance in CAD patients with DES implantation. The novel multiple risk score model was significantly associated with the risk of long-term ischemic events in these patients with an increment of scores. A meaningful improvement to predict adverse outcomes when multiple risk scores were applied to risk stratification. Further studies are needed to confirm these findings.

## Data Availability Statement

The raw data supporting the conclusions of this article will be made available by the authors without undue reservation. Further inquiries can be directed to the corresponding author.

## Ethics Statement

The studies involving human participants were reviewed and approved by Ethics Committee of General Hospital of Northern Theater Command. The patients/participants provided their written informed consent to participate in this study.

## Author Contributions

MQ designed a statistical plan and verified the underlying data, and was in charge of manuscript writing and statistical analysis. YL designed a statistical plan and verified the underlying data. YL, KN, ZQ, SM, HZ, XX, JL, KX, and XW contributed to recruiting subjects during the whole period of study. YH contributed to the leadership of the whole process of study conduction and acted as the key role of initiating, designing, conducting, and concluding the study. All authors contributed to the article and approved the submitted version.

## Funding

The I-LOVE-IT 2 trial was sponsored by Essen Technology (Beijing, China), and our study was also supported by the National Key Research and Development Program of China (2016YFC1301300 and 2016YFC1301303).

## Conflict of Interest

The authors declare that the research was conducted in the absence of any commercial or financial relationships that could be construed as a potential conflict of interest. The reviewer JT declared a shared affiliation, with two of the authors ZQ and JL to the handling editor at the time of the review.

## Publisher's Note

All claims expressed in this article are solely those of the authors and do not necessarily represent those of their affiliated organizations, or those of the publisher, the editors and the reviewers. Any product that may be evaluated in this article, or claim that may be made by its manufacturer, is not guaranteed or endorsed by the publisher.
