# Supplementary material for: A Novel Multiple Risk Score Model for Prediction of Long-Term Ischemic Risk in Patients With Coronary Artery Disease Undergoing Percutaneous Coronary Intervention: Insights From the I-LOVE-IT 2 Trial
Source: Front Cardiovasc Med. 2022 Jan 13;8:756379. doi: 10.3389/fcvm.2021.756379 (PMC8793781; doi:10.3389/fcvm.2021.756379)
Supplement: Supplementary file 1 [file Data_Sheet_1.docx]

A Novel Multiple Risk Score Model for Prediction of Long-term Ischemic Risk in Patients with Coronary Artery Disease Undergoing Percutaneous Coronary Intervention: Insights from the I-LOVE-IT 2 Trial

Supplementary material

This appendix complements the main manuscript and provides the results from additional analyses.

**Appendix S1. Risk score calculations**

GRACE score^1^ was calculated to estimate risk of death based on age, history of congestive heart failure, history of myocardial infarction, resting heart rate, systolic blood pressure, ST-segment depression, initial serum creatinine, elevated cardiac enzymes, and in-hospital percutaneous coronary intervention. The GRACE score was calculated by a web calculator ^2^.

ACEF score^3^ was calculated based on age, creatinine and ejection fraction to predict all-cause mortality. The ACEF was calculated using the formula that ACEF score = age/left ventricular ejection fraction +1 (if serum creatinine was≥2.0mg/dL).

Baseline SYNTAX score^4^ is a tool developed in connection with the SYNTAX Trial, a trial comparing PCI and Cardiac Surgery in complex, which is a comprehensive angiographic scoring system that is derived entirely from the coronary anatomy and lesion characteristics.

Residual SYNTAX score^5^ was calculated based on the remaining obstructive coronary disease after treatment with PCI.

The baseline and residual SYNTAX score were both calculated by an online web calculator^6^.

**Reference**

1. Eagle KA, Lim MJ, Dabbous OH, Pieper KS, Goldberg RJ, Van de Werf F, et al. A validated prediction model for all forms of acute coronary syndrome: estimating the risk of 6-month postdischarge death in an international registry. JAMA. (2004) 291:2727–33. doi: 10.1001/jama.291.22.2727

2. https://www.outcomes-umassmed.org/grace/acs_risk/acs_risk_content.html

3. Ranucci M, Castelvecchio S, Menicanti L, Frigiola A, Pelissero G. Risk of assessing mortality risk in elective cardiac operations: age, creatinine, ejection fraction, and the law of parsimony. Circulation. (2009) 119:3053–61. doi: 10.1161/CIRCULATIONAHA.108.842393

4. Sianos G, Morel MA, Kappetein AP et al. The SYNTAX Score: an angiographic tool grading the complexity of coronary artery disease. EuroIntervention : journal of EuroPCR in collaboration with the Working Group on Interventional Cardiology of the European Society of Cardiology 2005;1:219-27

5. Genereux P, Palmerini T, Caixeta A, Rosner G, Green P, Dressler O, et al. Quantification and impact of untreated coronary artery disease after percutaneous coronary intervention: the residual SYNTAX (Synergy Between PCI with Taxus and Cardiac Surgery) score. J Am Coll Cardiol. (2012) 59:2165–74. doi: 10.1016/j.jacc.2012.03.010

6. http://syntaxscore.org/calculator/start.htm

**Table S1. Clinical outcomes for patients excluded from the analysis**

| Outcome | (N=530) |
| --- | --- |
| Ischemic Events | 47(8.79%) |
| All-cause mortality | 20(3.74%) |
| Cardiac death | 11(2.06%) |
| All MI | 24(4.49%) |
| Target Vessel MI | 19(3.55%) |
| Stroke | 18(3.36%) |
| Definite/probable ST | 4(0.75%) |

MI=myocardial infarction; ST= stent thrombosis

**Table S2. Antiplatelet therapy during follow-up Stratified across Cumulative Risk Scores**

|  | 0 (N=514) | 1 (N=553) | 2 (N=632) | ≥3 (N=508) |
| --- | --- | --- | --- | --- |
| 6-month |  |  |  |  |
| Aspirin | 511 (99.42%) | 548 (99.10%) | (98.42%) | (97.05%) |
| P2Y12 inhibitor | 367 (71.40%) | 397 (71.79%) | 463 (73.26%) | 362 (71.26%) |
| 12-month |  |  |  |  |
| Aspirin | 506 (98.44%) | 543 (98.19%) | 618 (97.78%) | 488 (96.06%) |
| P2Y12 inhibitor | 202 (39.30%) | 232 (41.95%) | 268 (42.41%) | 187 (36.81%) |
| 24-month |  |  |  |  |
| Aspirin | 493 (95.91%) | 518 (93.67%) | 598 (94.62%) | 467 (91.93%) |
| P2Y12 inhibitor | 64 (12.45%) | 92 (16.64%) | 80 (12.66%) | 65 (12.80%) |
| 36-month |  |  |  |  |
| Aspirin | 475 (92.41%) | 504 (91.14%) | 570 (90.19%) | 438 (86.22%) |
| P2Y12 inhibitor | 33 (6.42%) | 59 (10.67%) | 51 (8.07%) | 27 (5.31%) |
| 48-month |  |  |  |  |
| Aspirin | 449 (87.35%) | 459 (83.00%) | 528 (83.54%) | 406 (79.92%) |
| P2Y12 inhibitor | 33 (6.42%) | 55 (9.95%) | 51 (8.07%) | 31 (6.10%) |

**Table S3. Multiple Imputation Results of 4-year Ischemic Events Stratified across Cumulative Risk Scores**

| _Imputation_ | RSs=0 | RSs=1 | RSs=2 | RSs=3 | RSs=4 | P for trend | HR (95%CI) | P value |
| --- | --- | --- | --- | --- | --- | --- | --- | --- |
| 1 | 6.21%(40/644) | 9.22%(60/651) | 11.92%(97/814) | 14.86%(59/397) | 19.48%(45/231) | <.0001 | 1.34(1.23-1.47) | <.0001 |
| 2 | 6.11%(39/638) | 8.91%(59/662) | 12.1%(98/810) | 15.21%(61/401) | 19.47%(44/226) | <.0001 | 1.36(1.24-1.49) | <.0001 |
| 3 | 6.56%(42/640) | 8.50%(56/659) | 12.05%(98/813) | 14.94%(59/395) | 20.00%(46/230) | <.0001 | 1.35(1.24-1.48) | <.0001 |
| 4 | 6.10%(39/639) | 8.99%(59/656) | 12.09%(99/819) | 15.27%(60/393) | 19.13%(44/230) | <.0001 | 1.35(1.24-1.48) | <.0001 |
| 5 | 6.23%(40/642) | 8.87%(58/654) | 12.13%(99/816) | 15.11%(60/397) | 19.30%(44/228) | <.0001 | 1.35(1.23-1.48) | <.0001 |
| 6 | 6.25%(40/640) | 8.85%(58/655) | 11.98%(98/818) | 15.74%(62/394) | 18.70%(43/230) | <.0001 | 1.35(1.23-1.48) | <.0001 |
| 7 | 6.18%(40/647) | 8.76%(57/651) | 12.31%(99/804) | 15.14%(61/403) | 18.97%(44/232) | <.0001 | 1.35(1.23-1.48) | <.0001 |
| 8 | 5.95%(38/639) | 9.13%(60/657) | 12.25%(100/816) | 14.82%(59/398) | 19.38%(44/227) | <.0001 | 1.35(1.24-1.48) | <.0001 |
| 9 | 5.95%(38/639) | 9.64%(64/664) | 11.93%(97/813) | 14.36%(56/390) | 19.91%(46/231) | <.0001 | 1.35(1.23-1.47) | <.0001 |
| 10 | 6.24%(40/641) | 8.81%(58/658) | 12.07%(98/812) | 15.29%(61/399) | 19.38%(44/227) | <.0001 | 1.35(1.24-1.48) | <.0001 |
| 11 | 6.37%(41/644) | 8.66%(56/647) | 12.32%(101/820) | 14.68%(58/395) | 19.48%(45/231) | <.0001 | 1.35(1.23-1.47) | <.0001 |
| 12 | 6.07%(39/643) | 9.44%(62/657) | 11.82%(96/812) | 14.94%(59/395) | 19.57%(45/230) | <.0001 | 1.35(1.23-1.47) | <.0001 |
| 13 | 6.23%(40/642) | 9.17%(60/654) | 11.95%(97/812) | 14.79%(59/399) | 19.57%(45/230) | <.0001 | 1.34(1.23-1.47) | <.0001 |
| 14 | 6.13%(39/636) | 9.15%(61/667) | 11.85%(96/810) | 15.44%(61/395) | 19.21%(44/229) | <.0001 | 1.35(1.23-1.48) | <.0001 |
| 15 | 6.17%(39/632) | 8.86%(59/666) | 12.01%(98/816) | 15.27%(60/393) | 19.57%(45/230) | <.0001 | 1.36(1.24-1.49) | <.0001 |
| 16 | 6.27%(40/638) | 8.80%(58/659) | 12.08%(98/811) | 15.00%(60/400) | 19.65%(45/229) | <.0001 | 1.35(1.24-1.48) | <.0001 |
| 17 | 6.03%(39/647) | 9.10%(59/648) | 12.21%(100/819) | 14.76%(58/393) | 19.57%(45/230) | <.0001 | 1.35(1.24-1.48) | <.0001 |
| 18 | 6.38%(41/643) | 8.65%(57/659) | 12.19%(99/812) | 15.62%(62/397) | 18.58%(42/226) | <.0001 | 1.35(1.23-1.47) | <.0001 |
| 19 | 6.36%(41/645) | 8.84%(58/656) | 12.21%(99/811) | 14.72%(58/394) | 19.48%(45/231) | <.0001 | 1.34(1.23-1.47) | <.0001 |
| 20 | 6.21%(40/644) | 8.90%(58/652) | 12.15%(99/815) | 14.82%(59/398) | 19.74%(45/228) | <.0001 | 1.35(1.24-1.48) | <.0001 |
| Overall | - | - | - | - | - | - | 1.35(1.23-1.48) | <0.001 |

The multiple imputation consisted of three steps. (1) Total 20 copies of the dataset were created, with the missing values replaced by imputed values. (2) The Cox regression analysis was performed to estimate the association of incremental number of risk scores with 48-month ischemic events in the each multiple imputed datasets. (3) The regression estimates from each imputed dataset were averaged together to produce overall estimated associations, with standard errors computed using Rubin’s rules, which take account of the variation in results across the imputed datasets.

**Table S4. Landmark Analysis of 4-year Ischemic Events Stratified across Cumulative Risk Scores**

|  | 0 (N=514) | 1 (N=553) | 2 (N=632) | ≥3 (N=508) | P for trend |
| --- | --- | --- | --- | --- | --- |
| Ischemic Events |  |  |  |  |  |
| 0 to 30 days | 11 (2.1%) | 16 (2.9%) | 27 (4.3%) | 39(7.68%) | <0.001 |
| 30 days to 4 years | 23 (4.5%) | 35 (6.3%) | 56 (8.9%) | 47(9.3%) | <0.001 |
| All-cause mortality |  |  |  |  |  |
| 0 to 30 days | 0 (0.0%) | 0 (0.0%) | 1 (0.2%) | 3(0.6%) | 0.02 |
| 30 days to 4 years | 11 (2.1%) | 13 (2.4%) | 20 (3.2%) | 29(5.7%) | 0.001 |

**Table S5. The sensitive analysis for primary endpoint**

| Ischemic Events | No. of Risk Scores Met the Individual Thresholds | | | | P value for trend | P value for interaction |
| --- | --- | --- | --- | --- | --- | --- |
|  | 0 | 1 | 2 | ≥3 |  |  |
| Treatment arms |  |  |  |  |  |  |
| BP-SES with 6-month DAPT  (N=726) | 7.27%  (12/165) | 9.19%  (17/185) | 12.32%  (25/203) | 13.29%  (23/173) | 0.04 | 0.33 |
| BP-SES with 12-month DAPT  (N=754) | 5.78%  (10/173) | 7.95%  (14/176) | 11.71%  (26/222) | 18.58%  (34/183) | <0.001 |  |
| DP-SES with 12-month DAPT  (N=727) | 6.82%  (12/176) | 10.42%  (20/192) | 15.46%  (32/207) | 19.08%  (29/152) | <0.001 |  |
| Presentation at admission |  |  |  |  |  |  |
| STEMI (N=276) | 6.38%  (3/47) | 3.23%  (2/62) | 19.10%  (17/89) | 14.10%  (11/78) | 0.03 | 0.55 |
| NSTEMI (N=231) | 7.14%  (3/42) | 10.64%  (5/47) | 14.06%  (9/64) | 23.08%  (18/78) | 0.01 |  |
| UA (N=1334) | 6.45%  (22/341) | 10.26%  (35/341) | 13.48%  (50/371) | 16.01%  (45/281) | <0.001 |  |

BP-SES, biodegradable polymer sirolimus-eluting stent; DP-SES, durable polymer sirolimus-eluting stent

**Table S6. Reclassification analysis for 4-year ischemic events**

|  | | Model with combined with 4 risk scores | | | |
| --- | --- | --- | --- | --- | --- |
|  |  | Low tertile  (<12%) | Moderate tertile  (12-18%) | High tertile  (>18%) | Reclassified % |
| Patients without events | | | | | |
| SYNTAX score | Low tertile  (<12%) | 1133 | 247 | 10 | 18 |
|  | Moderate tertile  (12-18%) | 158 | 281 | 66 | 44 |
|  | High tertile  (>18%) | 1 | 24 | 33 | 43 |
| Patients with events | | | | | |
| SYNTAX score | Low tertile  (<12%) | 111 | 46 | 4 | 31 |
|  | Moderate tertile  (12-18%) | 14 | 45 | 19 | 42 |
|  | High tertile  (>18%) | 0 | 5 | 10 | 33 |
| NRI: 12.5% (5.3%-20.0%), P=0.001 | | | | | |

|  | | Model with combined with 4 risk scores | | | |
| --- | --- | --- | --- | --- | --- |
|  |  | Low tertile  (<12%) | Moderate tertile  (12-18%) | High tertile  (>18%) | Reclassified % |
| Patients without events | | | | | |
| Residual  SYNTAX score | Low tertile  (<12%) | 1171 | 353 | 31 | 25 |
|  | Moderate tertile  (12-18%) | 119 | 181 | 48 | 48 |
|  | High tertile  (>18%) | 2 | 18 | 30 | 40 |
| Patients with events | | | | | |
| Residual  SYNTAX score | Low tertile  (<12%) | 114 | 56 | 11 | 37 |
|  | Moderate tertile  (12-18%) | 11 | 33 | 13 | 42 |
|  | High tertile  (>18%) | 0 | 7 | 9 | 44 |
| NRI: 9.4% (2.0%-16.8%), P=0.01 | | | | | |

|  | | Model with combined with 4 risk scores | | | |
| --- | --- | --- | --- | --- | --- |
|  |  | Low tertile  (<12%) | Moderate tertile  (12-18%) | High tertile  (>18%) | Reclassified % |
| Patients without events | | | | | |
| ACEF score | Low tertile  (<12%) | 1230 | 394 | 47 | 26 |
|  | Moderate tertile  (12-18%) | 58 | 132 | 34 | 41 |
|  | High tertile  (>18%) | 4 | 26 | 28 | 52 |
| Patients with events | | | | | |
| ACEF score | Low tertile  (<12%) | 111 | 73 | 15 | 44 |
|  | Moderate tertile  (12-18%) | 14 | 20 | 10 | 55 |
|  | High tertile  (>18%) | 0 | 3 | 8 | 27 |
| NRI: 12.1% (4.5-19.7%), P=0.002 | | | | | |

|  | | Model with combined with 4 risk scores | | | |
| --- | --- | --- | --- | --- | --- |
|  |  | Low tertile  (<12%) | Moderate tertile  (12-18%) | High tertile  (>18%) | Reclassified % |
| Patients without events | | | | | |
| GRACE score | Low tertile  (<12%) | 1053 | 177 | 7 | 15 |
|  | Moderate tertile  (12-18%) | 239 | 356 | 81 | 47 |
|  | High tertile  (>18%) | 0 | 19 | 21 | 48 |
| Patients with events | | | | | |
| GRACE score | Low tertile  (<12%) | 97 | 32 | 2 | 26 |
|  | Moderate tertile  (12-18%) | 28 | 63 | 23 | 45 |
|  | High tertile  (>18%) | 0 | 1 | 8 | 11 |
| NRI: 10.7% (3.3-18.1%), P=0.002 | | | | | |


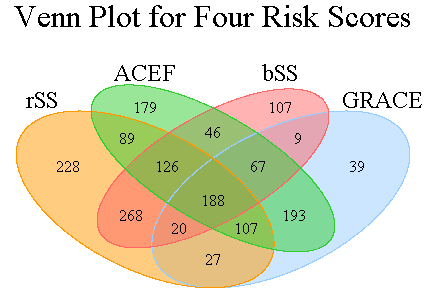


**Figure S1. The Venn diagram to demonstrate the coexistence of conditions of baseline SYNTAX score, residual SYNTAX score, ACEF score, and GRACE score**


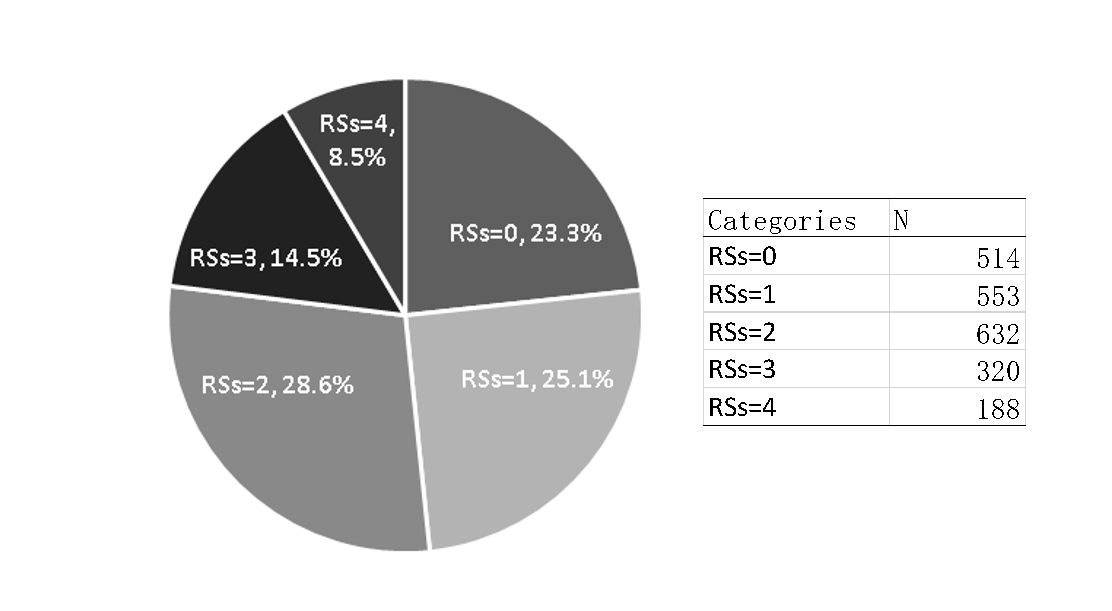


**Figure S2. The Distribution of Patients across the Cumulative Risk-score Categories**


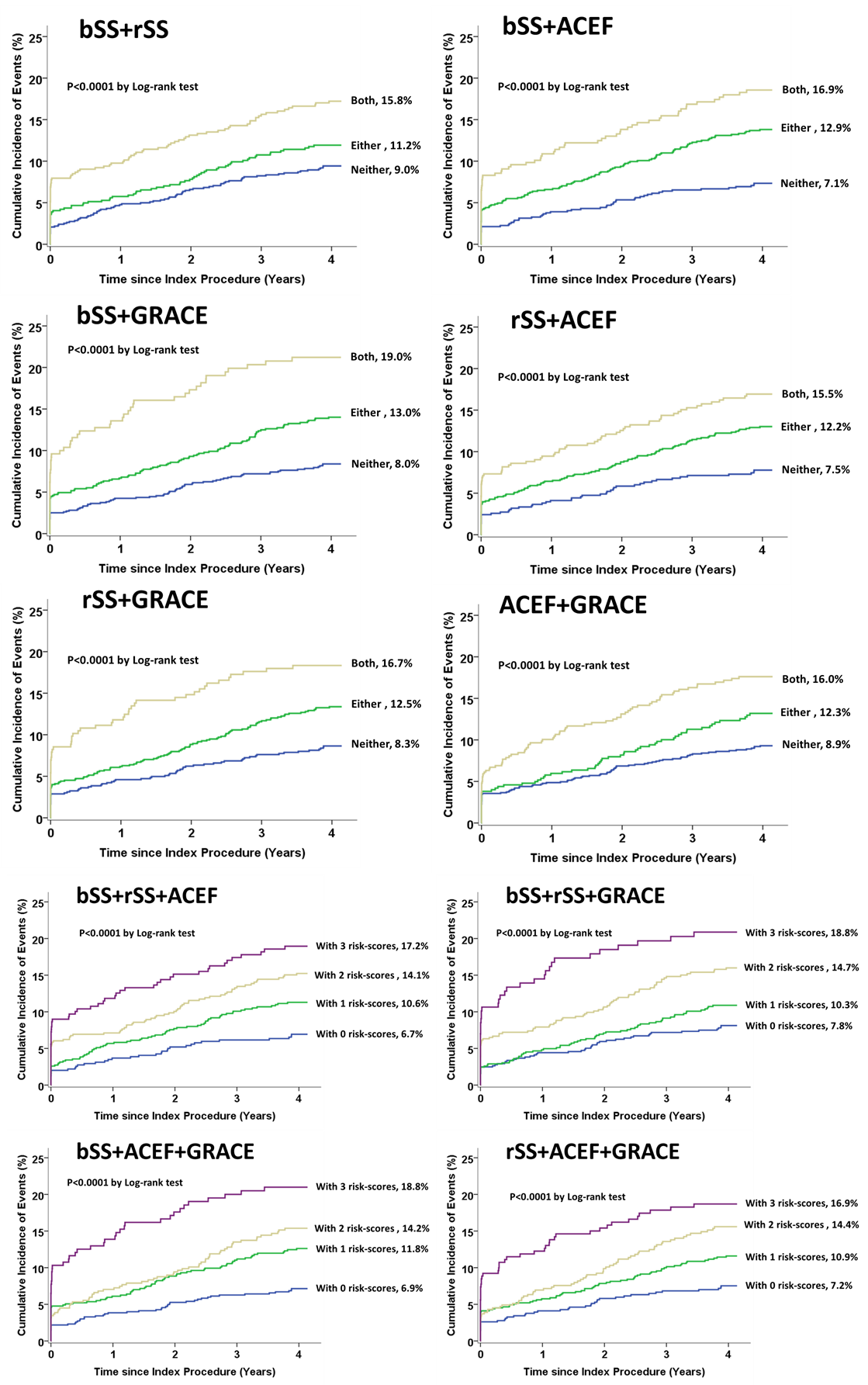


**Figure S3. Kaplan–Meier curves during Follow-up for 48-month Ischemic Events Among the Various Cumulative Risk-score Categories.**


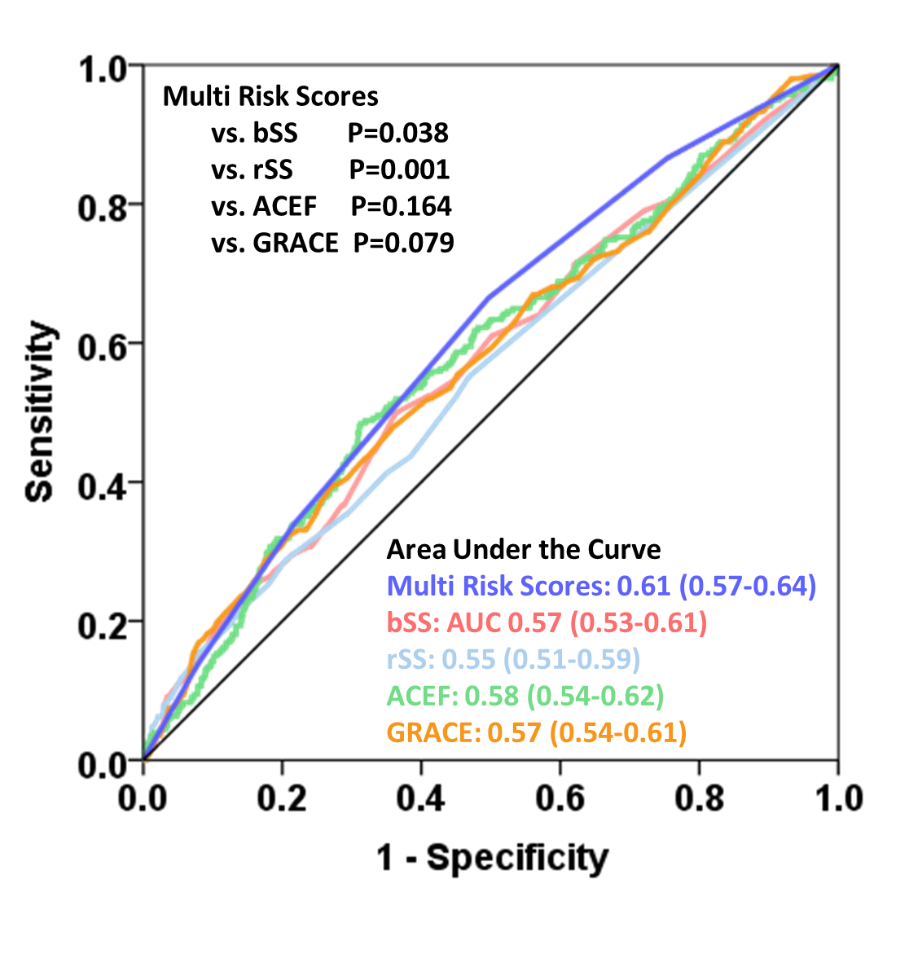


**Figure S4. Receiver operating characteristic (ROC) curves of incremental number of risk scores, baseline SYNTAX score, residual SYNTAX score, ACEF score, and GRACE score for 48-month ischemic events**
